# Validation of a graphic test to quantitatively assess the dominant hand dexterity

**DOI:** 10.1371/journal.pone.0271889

**Published:** 2022-08-01

**Authors:** Alessandra Angelucci, Andrea Tettamanti, Elisabetta Sarasso, Massimo Filippi, Andrea Aliverti, Marina Scarlato

**Affiliations:** 1 Dipartimento di Elettronica, Informazione e Bioingegneria, Politecnico di Milano, Milan, Italy; 2 Rehabilitation Department, IRCCS San Raffaele Scientific Institute, Milan, Italy; 3 Neurology Unit, Neurorehabilitation Unit, Neurophysiology Service and Neuroimaging Research Unit, IRCCS San Raffaele Scientific Institute, Milan, Italy; 4 Vita-Salute San Raffaele University, Milan, Italy; Universitat de les Illes Balears, SPAIN

## Abstract

Dexterity dysfunction is a key feature of disability in many neurological and non-neurological diseases. The Nine-Hole Peg Test (NHPT) is the most used test to assess hand dexterity in clinical practice but presents limitations. A new graphic test to enhance objective evaluation of the of the dominant hand dexterity is proposed. The task consists in drawing a continuous line in paths composed by a part with multiple orthogonal changes of direction (‘meander’), and a second part derived from the Archimedean spiral (‘spiral’). The test was validated in 200 healthy controls and 93 neurological patients. 48 patients performed also the NHPT. Several parameters were analyzed, among which total time, total length, number of touches and number of crossings. Healthy subjects display statistically significant differences with respect to pathological subjects in the case of total time, number of touches, and number of crossings (p<0.001), but not in the case of total length (p = 0.27) needed to complete the second sheet. Moreover, healthy controls display a learning effect, the time needed to complete the second sheet was significantly lower than for the first sheet (p<0.001), and an inverse correlation with age was observed (r = 0.56, p<0.001). The comparison between the NHPT and the new test showed a strong positive correlation (r = 0.71, p<0.001) whereas touches and crossing a weak positive one (r = 0.35, p = 0.01). The new test distinguishes between a slow but precise performance and a fast but imprecise performance, thus providing additional information with respect to NHPT.

## 1. Introduction

The hand is the most versatile manipulative organ, and a fine integration of manual dexterity and visual-motor coordination is required in order to perform a complex task, as writing or drawing.

Hand dexterity has been defined scientifically as “the ability to adequately solve any motor task precisely, quickly, rationally and deftly” [1] where flexibility with respect to the changing environment is an important feature [2]. Hand dexterity plays a larger role in evaluating fine motor disability and it is a human beings distinctive feature. It can undergo many alterations due to aging, traumas and orthopedic or neurological disorders, which result in several limitations during daily life activities.

Among the main causes, neurological diseases can affect the mechanism underlying fine motor ability in different ways, modifying muscle force and/or coordination, and/or movement regulation and/or visual and sensory impairment. Moreover, in neurological diseases such as multiple sclerosis or spinocerebellar ataxias involvement of different neuroanatomical pathways can coexist resulting in an even more complex impact on manual dexterity. For example, regarding grip force, it has been already demonstrated that subjects with stroke have an impaired proactive grip force adjustments whereas subjects with cerebellar dysfunction have a defective grip–load force coordination and subjects with diseases involving basal ganglia have difficulties in grip-load force regulation [3]. Therefore, it is important to evaluate and quantify fine motor disability in a patient in order to try to define the deficit, to establish a baseline and to evaluate the disease progression.

There are several tests already in use in the clinical practice to assess hand dexterity, and they can be divided into three groups. In the first one there are tests evaluating functionally several activities to assess the upper limbs performance. The first group comprises the Fugl-Meyer Assessment (FMA) [4], the Wolf Motor Function Test (WMFT), and the Action Research Arm Test [5]. The tests belonging to the second ground consist in time trials based on a simple task that need to be completed. The second group comprises the Nine-Hole Peg Test (NHPT) [6], the Buttoning Test (BT) [7], the Box and Block Test (BBT) [8], the Minnesota Manual Dexterity Test (MMDT), the Workability Rate of Manipulation Test [9], and the Twenty-Five-Hole Peg Test (TFHPT) [10]. Finally, the tests of the third group evaluate the condition of the upper limb functionality with a questionnaire administered to the patients. Examples of tests of the third group are the Motor Activity Log [11], and the Disability of the Arm, Shoulder and Hand (DASH) [12].

Among the tests listed, the NHPT is a standardized, quantitative assessment used to measure finger dexterity and by far the most used in the clinical practice [13] due to execution speed, easy and inexpensive equipment required and no need of any prior training. This test is administered by asking the patient to take the pegs from a container, one by one, and place them into the holes on a board with 9 holes, as quickly as possible. Participants must then remove the pegs from the holes, one by one, and replace them back into the container. Scores are based on the time taken to complete the test activity, recorded in seconds; there is a time limit of 300 seconds to complete the test. Normative data have been proposed in the literature in 1985 [6] and updated in 2003 [14]. Recently [15], a comprehensive study involving 4319 healthy subjects showed better performance by dominant hands and females but a decrease achievement across the age span.

There are however several limitations of the NHPT. Once the test has been performed, only a score remains, without further possibility to review exactly how the patient did in each phase of the test; it is not possible to distinguish between subjects that are slow but precise and subjects that take more time due to imprecisions or difficulties in performing the task itself. Finally, the presence of a time limit introduces a floor effect on people with severe upper limb dysfunction [16].

The aim of the present work is to overcome the main limitations of the NHPT proposing a new test enabling quantitative parametrization of temporal and spatial performances. A new graphic test called SpAcCo (Speed- Accuracy- Coordination) test has been developed, validated and here proposed.

A distinction between speed of execution and precision is clinically and functionally relevant. Another limitation that this work seeks to overcome is the floor effect, which as of today does not allow a proper evaluation of hand dexterity in severely impaired patient populations.

## 2. Materials and methods

### Patient recruitment

Potential participants were pre-screened via an informal verbal conversation to determine eligibility. The inclusion criteria were that participants had to be 18 or older with no blindness or low vision reported neither muscular skeletal, arthritis or neurological problems. Subjects with hand fingers amputation or any limitation in holding and using a pen or difficulties in understanding the task required were excluded.

All participants gave written informed consent to participate in the study, and the study was approved by the Ethical Committee of Politecnico di Milano (protocol number 08/2020).

### SpAcCo test

**Fig 1:**
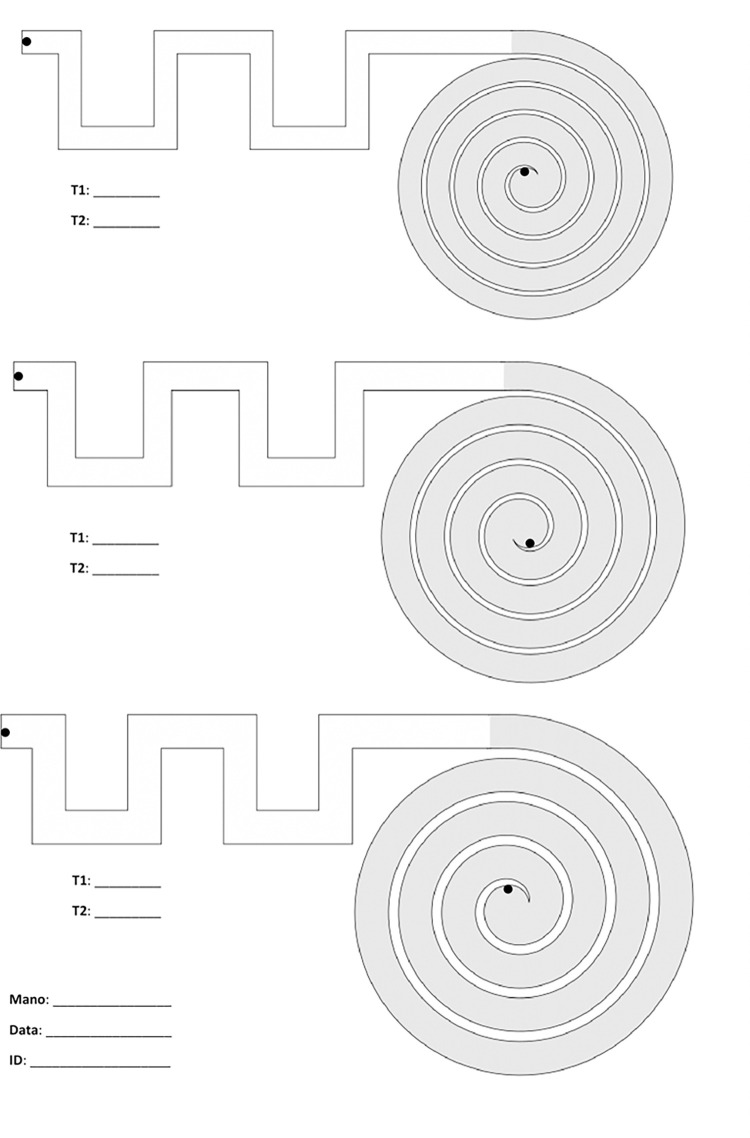
SpAcCo test: Three paths are present on each sheet, and they must be completed from the bottom (largest path) to the top (smallest path).

The SpAcCo test consists in asking the subject to connect the start and the end of a pre-defined path with a red line, on a paper sheet. Each sheet presents three paths with the same shape but different width, and the subject must repeat all three paths on two different sheets. Each path is composed of two traits: one is a meander-like part where there are multiple orthogonal changes of direction (‘meander’); the second trait is derived from the Archimedean spiral (‘spiral’). These two parts have different background colors to allow a clear distinction during the image processing phase. The subjects participating in the test must complete each path as fast as possible without touching the borders; an operator uses a chronometer to measure the time needed to complete each trait (meander and spiral) of the six paths (three paths per two sheets). There is no time limit to complete each path. The order of execution of the tests is always the same: the first path to follow is the one at the bottom of the sheet, i.e., the one with the greatest width, followed by the middle path with intermediate width and the path at the top of the sheet with the smallest width. This order is repeated for the first and the second sheet. An image of one empty sheet is shown in Fig 1. ‘T1’ and ‘T2’ indicate, respectively, the time needed to complete the meander and then the spiral. ‘Mano’ (Italian translation of ‘hand’) indicates the dominant hand. The date is also reported, so that the files can be archived and consulted in a future moment.

Upon completion of the test, the two sheets are scanned and a digital copy with.jpeg extension is created, thus allowing the creation of a digital archive comprising all the scanned tests. Each sheet is then analyzed with a MATLAB® software, presented in the next section.

### Software analysis

**Fig 2:**
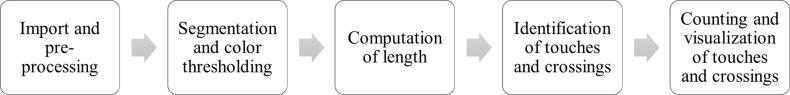
Steps followed during the software analysis.

The software analysis follows the steps shown in Fig 2.

After scanning, each image is imported into MATLAB® and resized into a 3507x2480 pixels image to compensate for the differences in resolution between different scanners. The digitized image presents imperfections due to intrinsic limitations of the quantization process that takes place when the image is scanned. Furthermore, there is a background noise on digitized images that causes some pixels to appear red even if they are not and the background not to be uniform. To compensate for these imperfections, multiple filtering passages are performed with the *Image Processing* MATLAB® toolbox: contrast enhancement, a 3x3 median filter, a Laplacian filter, and a 5x5 Gaussian filter.

**Fig 3:**
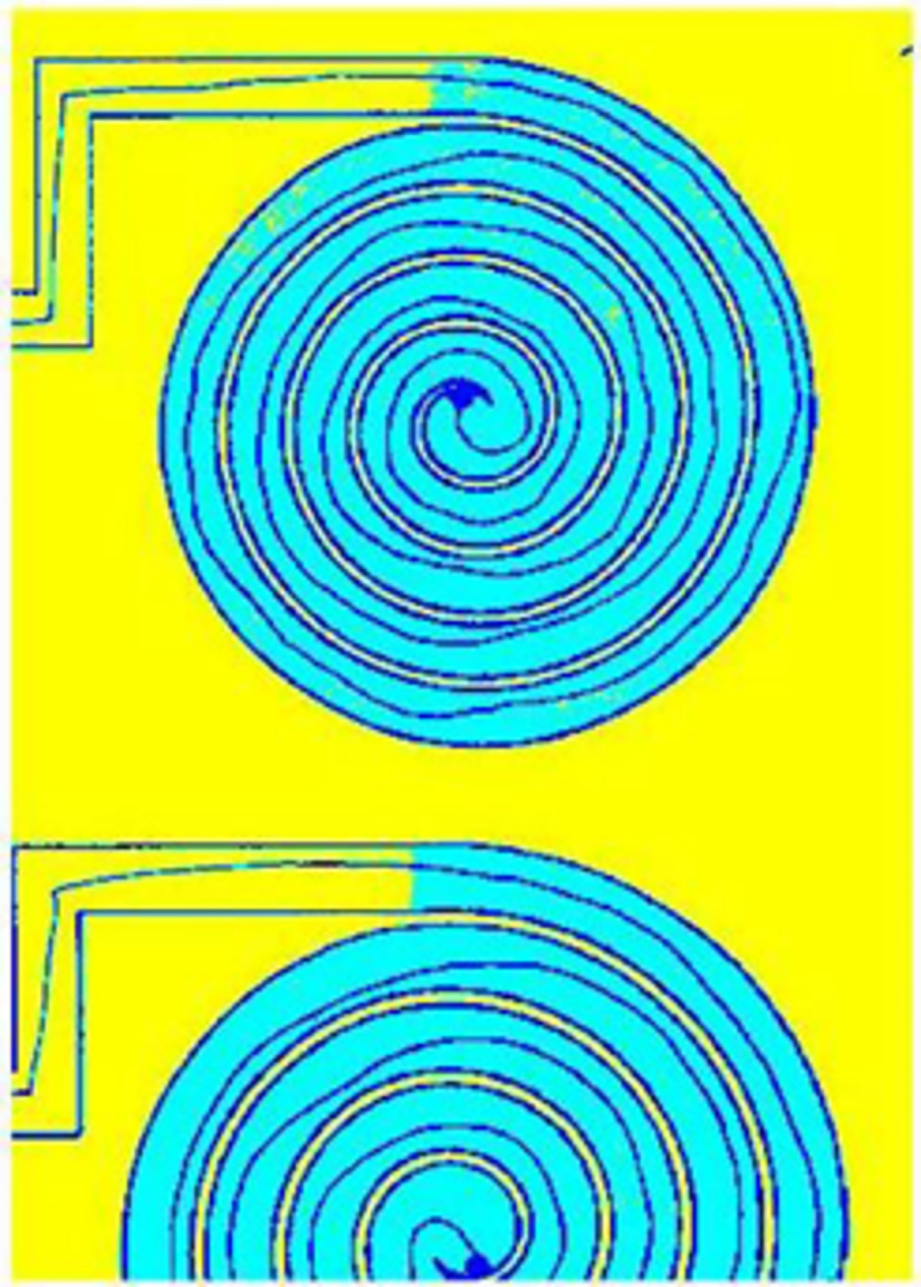
Image after the division into three intervals based on greyscale intensity. Test taken by a healthy control.

A criterion was imposed to categorize the image between three different intensities of grey. The actual scale of grey were divided in a selected number of intervals (in this case, 3 intervals) and all pixels in each interval were uniformed to the highest grey intensity of the interval. This division allowed to distinguish between the white background (colored in yellow), the borders of the path and the line drawn by the patient (colored in black) and the background of the spiral (colored in light blue). The output of this division into 3 intervals is shown in Fig 3; the colors have been inserted only for visualization purposes.

The second step consisted in distinguishing between the different paths on a sheet and the meander and the spiral of each path, to provide data paired with the time information.

After the division into three intervals based on greyscale intensity, a mask was created with the shape of the spiral, so that there would be an image only with the spiral data and its opposite only with the spiral data.

The Otsu method was exploited [17] to achieve a more accurate segmentation and to further prepare the image for length computation, which is based on the counting of red pixels. For each image the grayscale can be different, as images might be lighter or darker depending on the scanner output. For this reason, the thresholds have been determined for every image by first analyzing the histogram of the colormap and then choosing the thresholds of the grey intensities in a way that minimizes variance between classes, according to the previously cited Otsu method.

**Fig 4:**
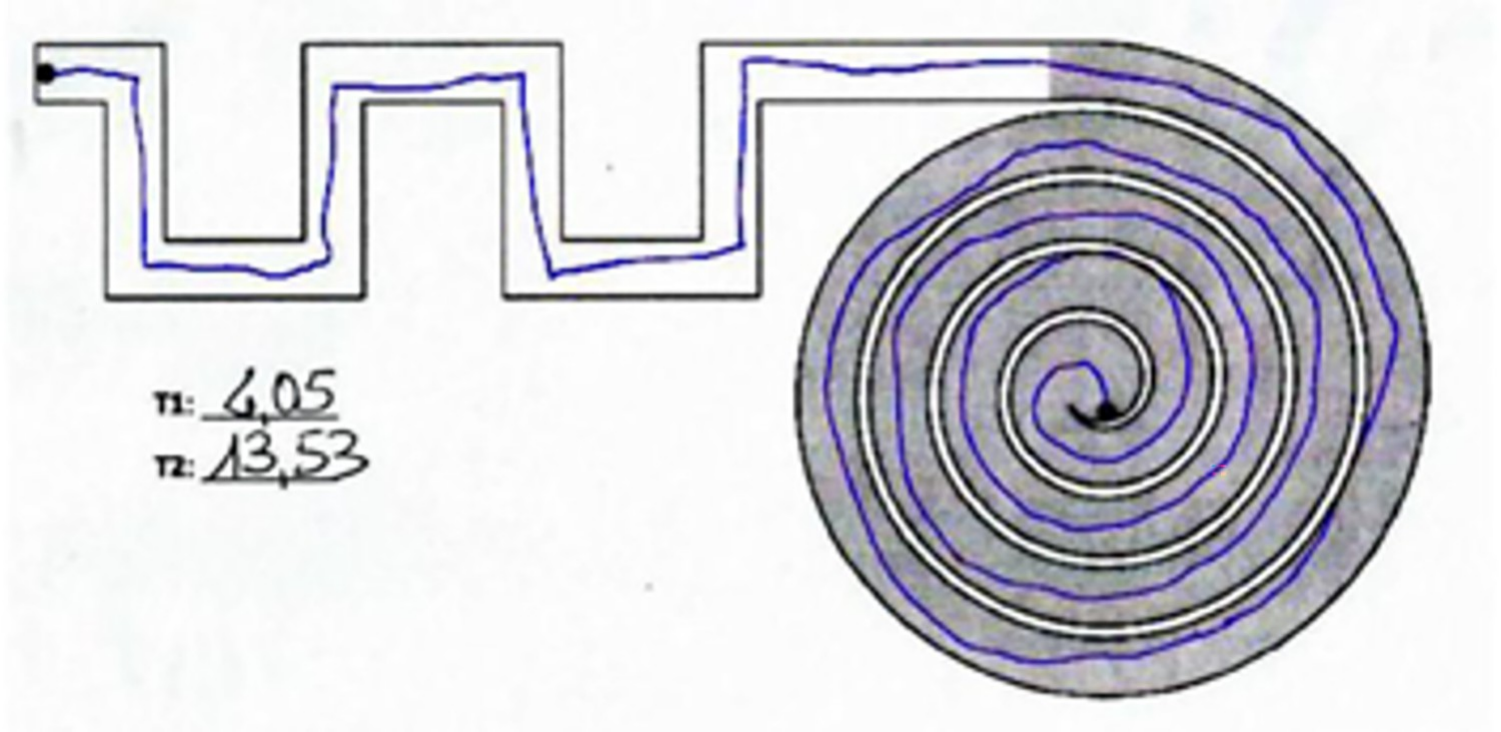
Recognized line colored in blue after the proper thresholding passages. Test taken by a healthy control.

The identification of the line and the subsequent computation of length was based on red pixels counting. The *Color Thresholder* app that is present in the *Image Processing Toolbox* was used to segment the image based on colors but also on the previous information obtained on the luminosity of the image itself (i.e., the thresholds to detect red pixels are different if the image is lighter or darker). For visualization purposes, the recognized red pixels are colored in blue as shown in Fig 4.

The most critical issue in determining the length of the line is the thickness of the line itself, which can vary between tests and therefore does not allow a direct conversion between the number of recognized red pixels and the length. For this reason, the thickness of the recognized trait was first reduced to a unit thickness of one pixel so that the variability was removed and only the shape remained (‘skeleton’).

By counting the pixels of the unit thickness in each of the six sections (spiral and meander for all three paths on a sheet), the value of length expressed in pixel was obtained. Since each scanned file had been resized into a 3507x2480 pixels image, it was possible to apply the same conversion factor to all results to provide a measurement of length in cm. The equation used is the following and is based on the dimensions of an A4 sheet:

$$Length\;[cm]=Length\;[pixels]∙\frac{21\;[cm]}{2480\;[pixels]}$$

**Fig 5:**
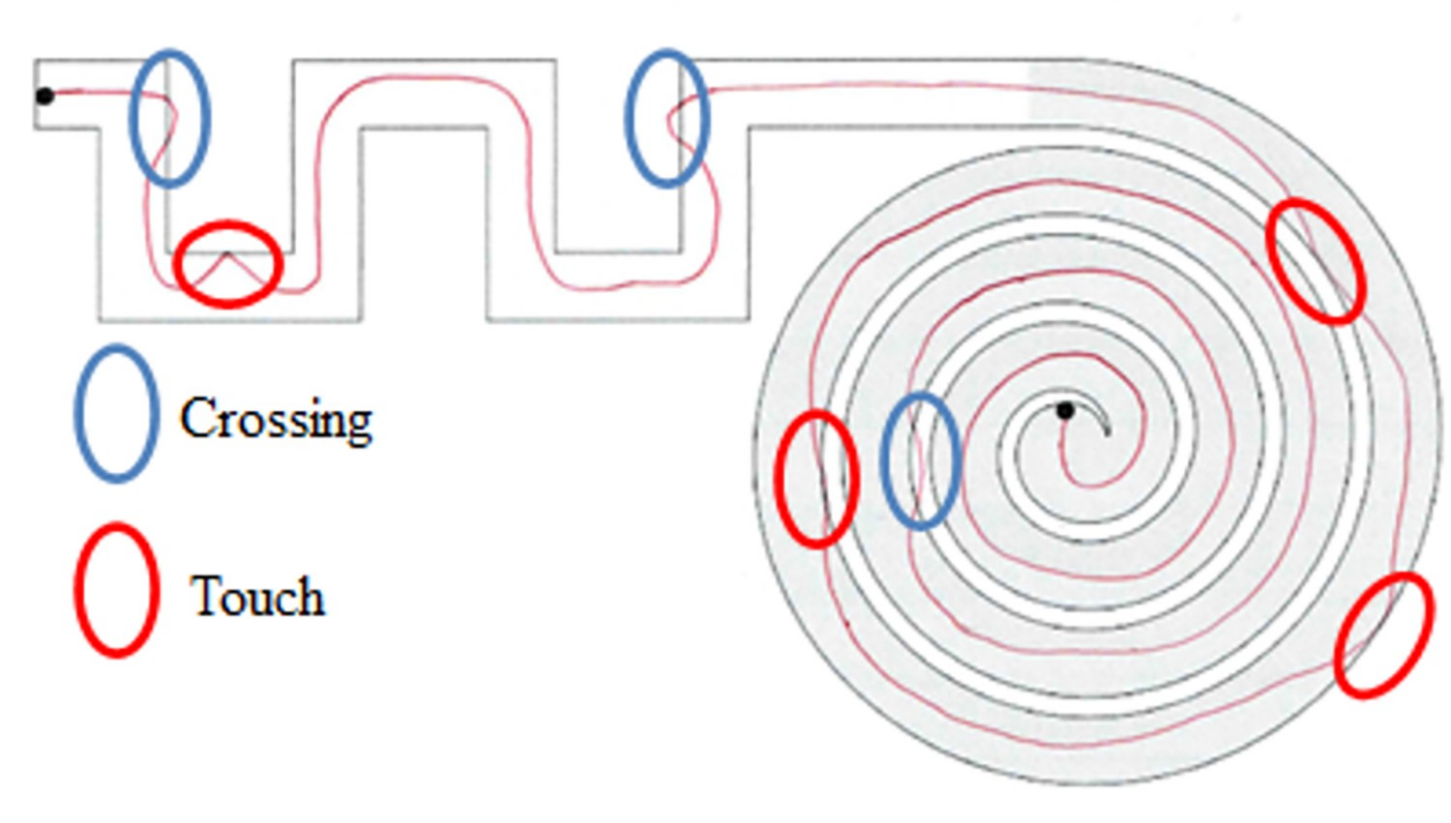
**Representation of touches (highlighted in red) and crossings (highlighted in blue).** The shown test was performed by the paper authors for visualization purposes and does not belong to the analyzed dataset.

Touches are here defined as the points in which the line drawn by the subject only touches the border of the path without stepping outside of it, while crossings are the moments in which the line passes from one side of the border to the other. An example is shown in Fig 5.

To identify touches, a mask was created with only the borders of each path. The mask was created for each single path starting from the image with the line colored in blue (shown in Fig 4) to account for the small differences between each digital image; the method to create the mask was the same used in the previous stages to segment the file into different sections using RGB color thresholds.

**Fig 6:**
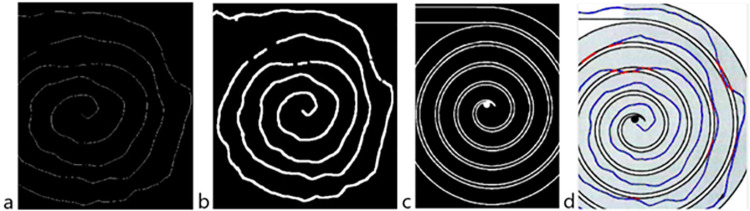
(a) ‘skeleton’ of the trait; (b) dilated trait with a thickness of 10 pixels; (c) mask of the path; (d) points where the dilated trait and the mask were found to be superimposed, highlighted in red. Test taken by a pathological subject.

The mask of the borders was then superimposed to the image of the unit trait (‘skeleton’) after the latter was dilated to a final thickness of 10 pixels. This was done to have a detectable superimposition between the mask and the final trait. After the two images were superimposed, a pixel-by-pixel control was performed to assess whether there were points in common between the two images and a new image was created, with the points of superimposition and the 3x3 matrix around them highlighted in red. The passages are illustrated in Fig 6.

Given that more than one pixel corresponds to a touch, the identified pixels have been clustered. Only clusters with 70 or more red pixels were counted as touches, while the smaller clusters were discarded as noise. The counting of touches is referred to each path and not divided between the meander and the spiral of each path. Thus, for each sheet three values of touches are available, one for the small path, one for the medium and one for the large.

To identify crossings, an algorithm like the one used for touches was applied. In this case, it was necessary to identify all that was outside the border; a binary image of the sheet was obtained, where the background was in black and the paths in white.

The skeleton of the line, this time without dilation, was superimposed with the binary image. After the two images were superimposed, a pixel-by-pixel control was performed to assess whether there were points in common between the two images and a new image was created. Only clusters with 250 or more pixels were identified as clusters, so that noise and the written measured times would be discarded.

### Statistical analysis

The statistical analysis was performed with an ad hoc software written in the Python programming language and based on the tools for statistics of the SciPy library [18].

In case of paired data sets, the following procedures were applied: first, the Shapiro-Wilk test was applied on the distributions to check for normality; in case of normal distributions, a paired t-test was performed, otherwise the Wilcoxon signed-rank test was used as alternative. Paired data sets were tested in terms of learning effect. To check for learning effects, the sums of times obtained in sheet one were compared with those obtained in sheet two in the case of healthy subjects.

Then, healthy subjects were compared to pathological subjects on their performances on the second sheet (time, length, touches, and crossings). After the Shapiro-Wilk test to check for normality, the tests chosen were the independent samples t-test for normal distributions and the Whitney U Test for non-normal distributions.

Also, in the case of correlation analysis, the first test to be applied was the Shapiro-Wilk to check for normality. In case of normal distributions, the Pearson correlation coefficient was computed; otherwise, the Spearman rank-order correlation coefficient was used. The correlation analysis was used (1) to assess whether the time to complete the second sheet and the age of the subject are correlated in the case of healthy subjects; (2) to check whether the NHPT results are correlated with time and/or with a metric of quality of execution, and if time and quality are correlated between each other. The parameter chosen for time was the time needed to complete the second sheet, while the parameter needed for quality was chosen as the sum of touches and crossings in the second sheet (‘errors’). The rationale behind this specific analysis was that a subject might be slow but precise or fast but imprecise, and the NHPT is not able to distinguish between slowness and quality of execution.

Finally, a Principal Component Analysis (PCA) was performed on the dataset. The input variables were age, total time, total length, number of touches, and number of crossings; the last four parameters were referred to the second sheet. The aim of this analysis was to assess whether the test could be able to detect the presence of a pathology without a priori knowledge. Data were standardized to have a mean equal to 0 and a variance equal to 1, then the explained variance of the principal components was plotted to choose only the components that could explain most of it. After choosing the appropriate number of principal components, data were plotted and divided by pathology for visual inspection.

## 3. Results

### Population

A total of 200 healthy controls (median age 45.5 ± 15.5, 99 men, 101 women) were tested. All healthy participants were recruited from the Ospedale San Raffaele employees or employees’ family members.

A total of 92 subjects (median age 26.5 ± 9.75, 36 men, 56 women) with a definitive diagnosis of genetic or neurodegenerative neurological disorders (Table 1) were recruited among the in- and out- patient service of the Ospedale San Raffaele Neurorehabilitation Unit.

**Table 1:** Characteristics of the participants (healthy control and patients). The last column reports the number of subjects that have performed the Nine-Hole Peg Test as well as the SpAcCo test.

Label	Pathology	Total number	NHPT
0	Healthy controls	200	0
1	Genetic ataxias	25	6
2	Extrapyramidal diseases	12	10
3	Muscular diseases	22	20
4	Nerve disorders	15	12
5	Other neurological diseases (OND)	18	0
Tot	Patients	92	48

Table 2 summarizes the median results obtained by healthy and pathological subjects. All parameters in this table comprise the results on the first and the second sheet without distinction. All the distributions analyzed were not normal according to the Shapiro-Wilk test.

**Table 2:** Median and Inter-Quartile Ranges (IQRs) of time, length, touches, and crossings in healthy and pathological subjects, divided by path size.

Population	Path	Time		Length		Touches		Crossings	
		Median	IQR	Median	IQR	Median	IQR	Median	IQR
**Healthy**	**Big**	18.4	9.9	73.6	4.0	0.0	0.5	0.0	0.5
	**Medium**	21.3	8.9	73.1	3.5	0.0	1.1	0.0	0.5
	**Small**	25.1	9.1	74.1	4.0	1.0	3.0	0.0	1.0
**Pathological**	**Big**	31.6	21.0	73.8	6.4	0.0	1.3	0.5	2.5
	**Medium**	32.3	20.4	73.5	7.8	1.5	9.1	1.0	3.0
	**Small**	37.3	20.9	73.7	6.2	2.0	6.6	1.5	3.1

The differences between the controls and patients regarding total time, total touches and total crossings are statistically significant according to the Mann-Whitney U Test (p<0.001 in all cases), but not those regarding total length (p = 0.27).

**Fig 7:**
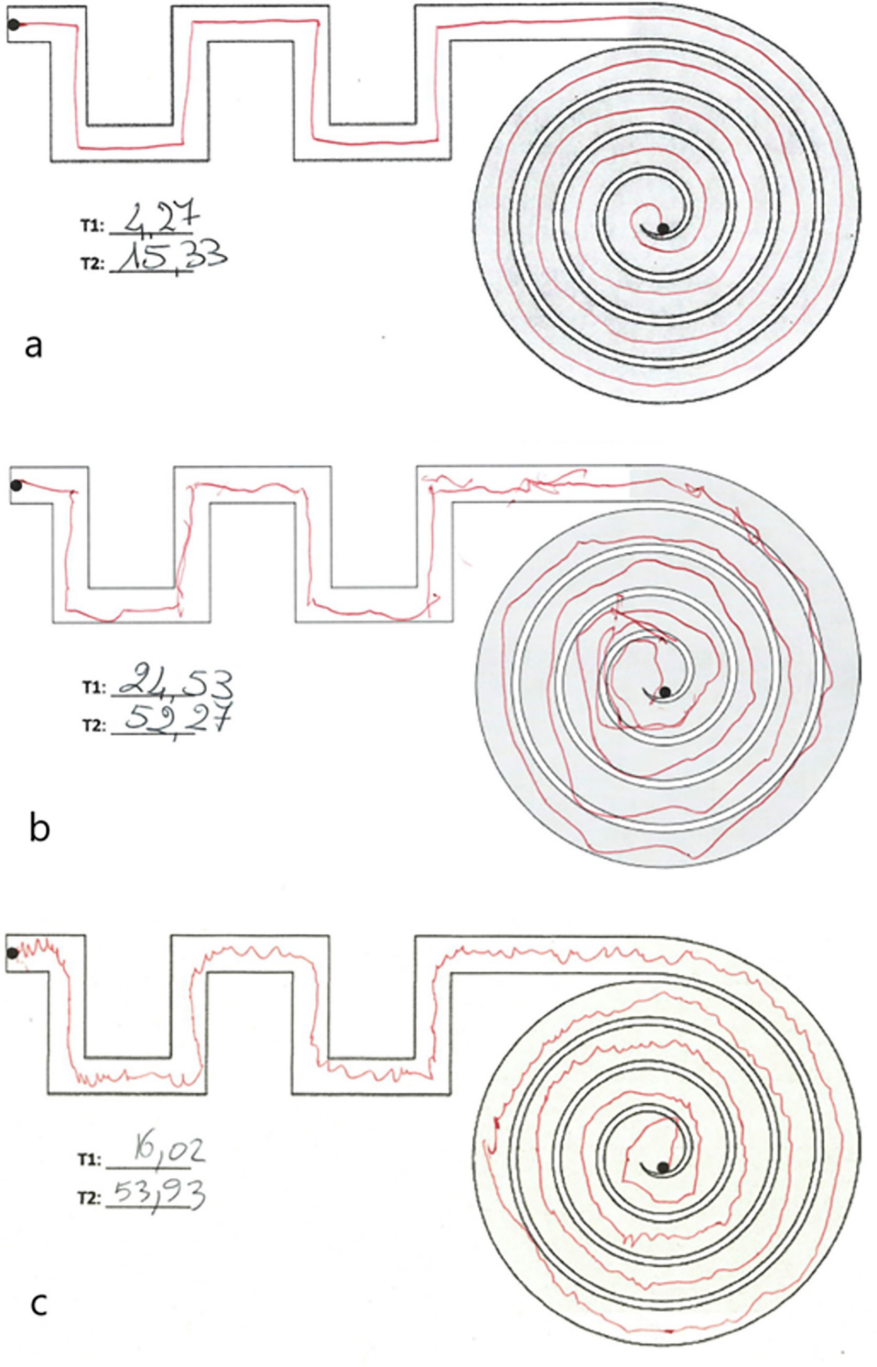
Medium path drawn by (a) a healthy subject; (b) an ataxic subject; (c) a subject with a hereditary peripheral neuropathy. The white background corresponds to the ‘meander’, while the grey background to the ‘spiral’ section of the path.

Moreover, visual inspection of graphical tests can provide insights on the performance of the subjects before and in addition to the quantitative analysis (Fig 7).

### Healthy controls: Correlation between time and age

**Fig 8:**
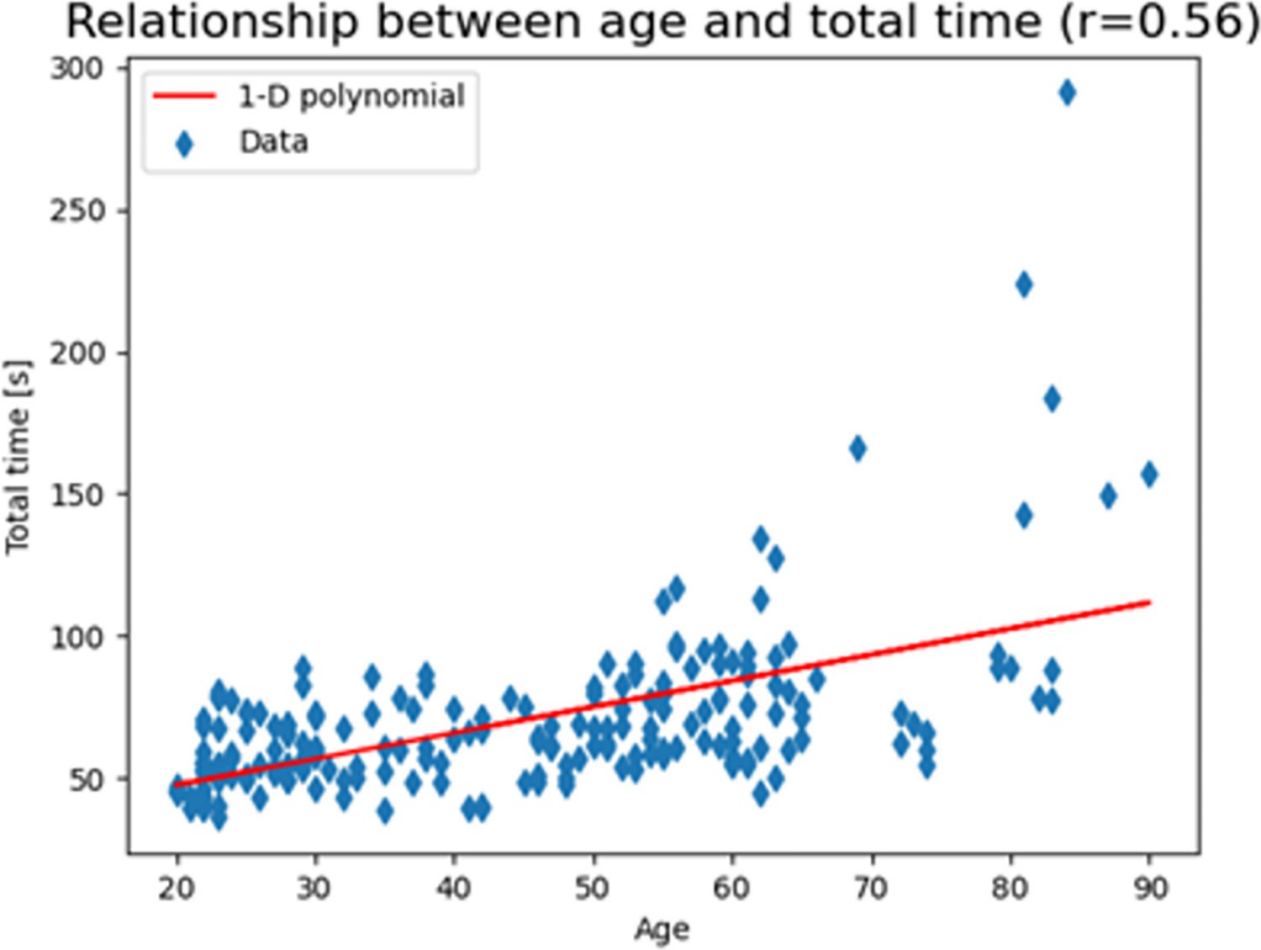
The scatter plot of the results with a 1-D polynomial trend line that clearly shows how total time increases with increasing age in healthy controls.

Only in the cohort of healthy control, a strong positive correlation was found (r = 0.56) with statistical significance (p-value < 0.001) between total time and age. The chosen parameter was the Spearman rank-order correlation coefficient due to absence of normal distribution (Fig 8).

### Healthy controls: Learning effect between sheets

**Fig 9:**
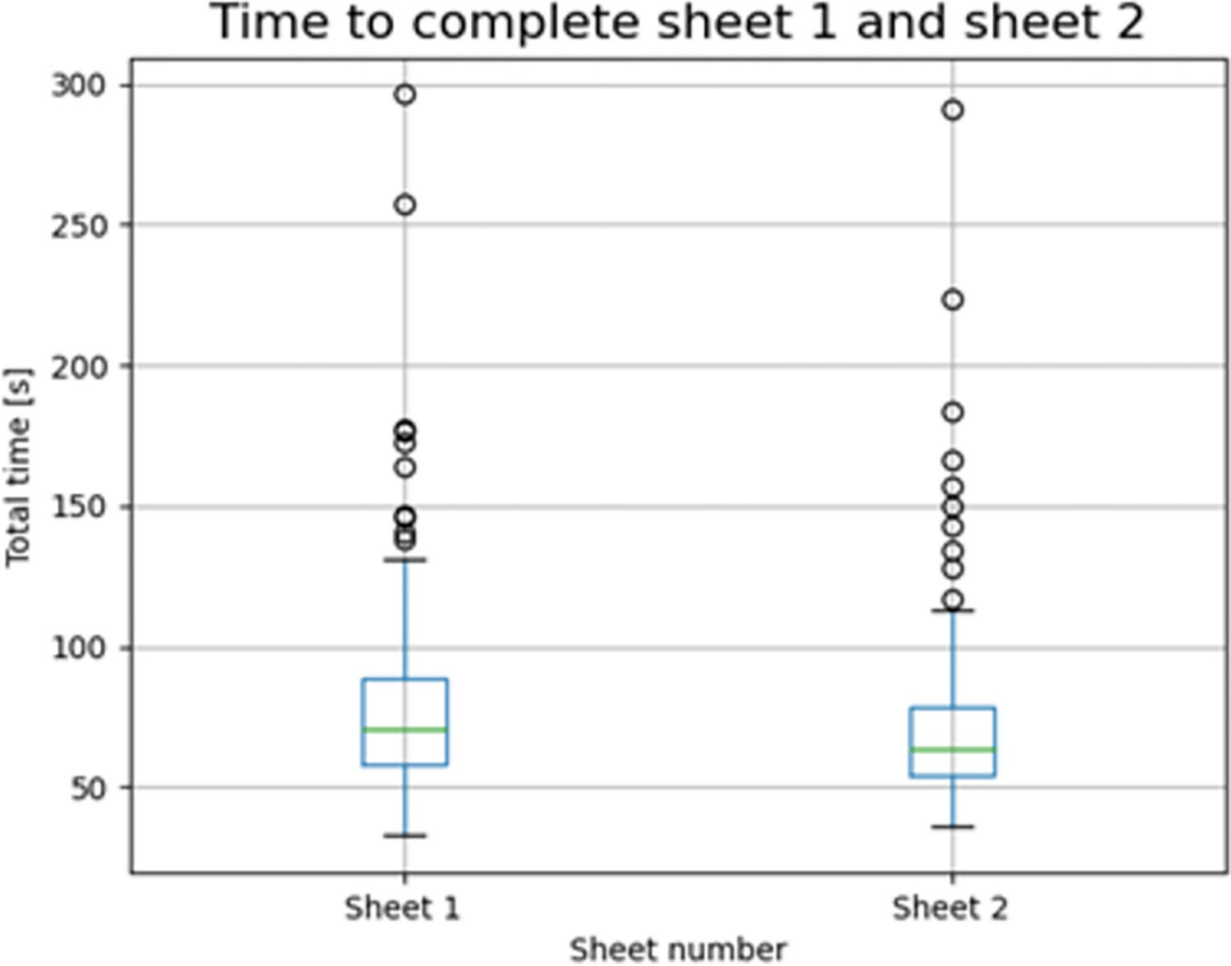
Time to complete the tests on the first and on the second sheet in healthy controls.

Only in the healthy control group, a learning effect was found already between the big and the medium path of the first sheet (p-value < 0.001) and an even more discrete time reduction was evident between the total time to complete the first and the second sheet (p-value < 0.001) indicating that task repetition despite the different width induces a time improvement (Fig 9). The mean time needed to complete the first sheet was 78 seconds, while it was 70 seconds for the second sheet.

### Correlation between SpAcCo and NHPT results

We compared the SpAcCo and the NHPT performance results in a sub cohort of neurological patients.

**Fig 10:**
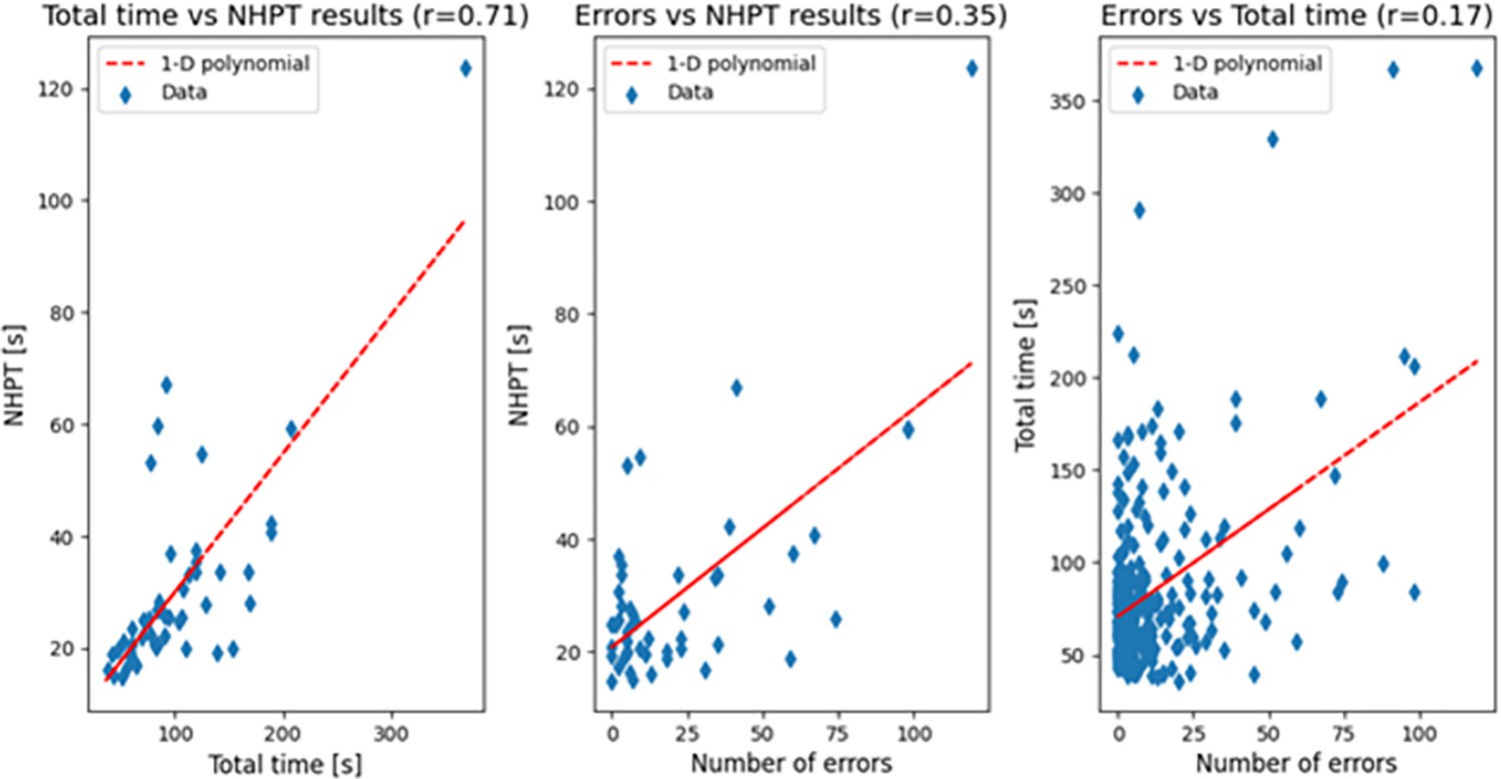
Correlation between total time to complete the second sheet and NHPT results (left), number of errors and NHPT results (middle), and number of errors and total time (right) in all pathological populations.

Total times and NHPT results showed a strong positive correlation (r = 0.71, p<0.001) whereas total errors (i.e., sums of touches and crossings) and NHPT results there is a weak positive correlation (r = 0.35, p = 0.01). This shows that the SpAcCo test can distinguish between a slow but precise performance and a fast but imprecise performance, thus providing an additional information with respect to NHPT results. The scatter plots of the results are shown in Fig 10 together with the respective 1-D polynomial trend line.

### Principal component analysis

**Fig 11:**
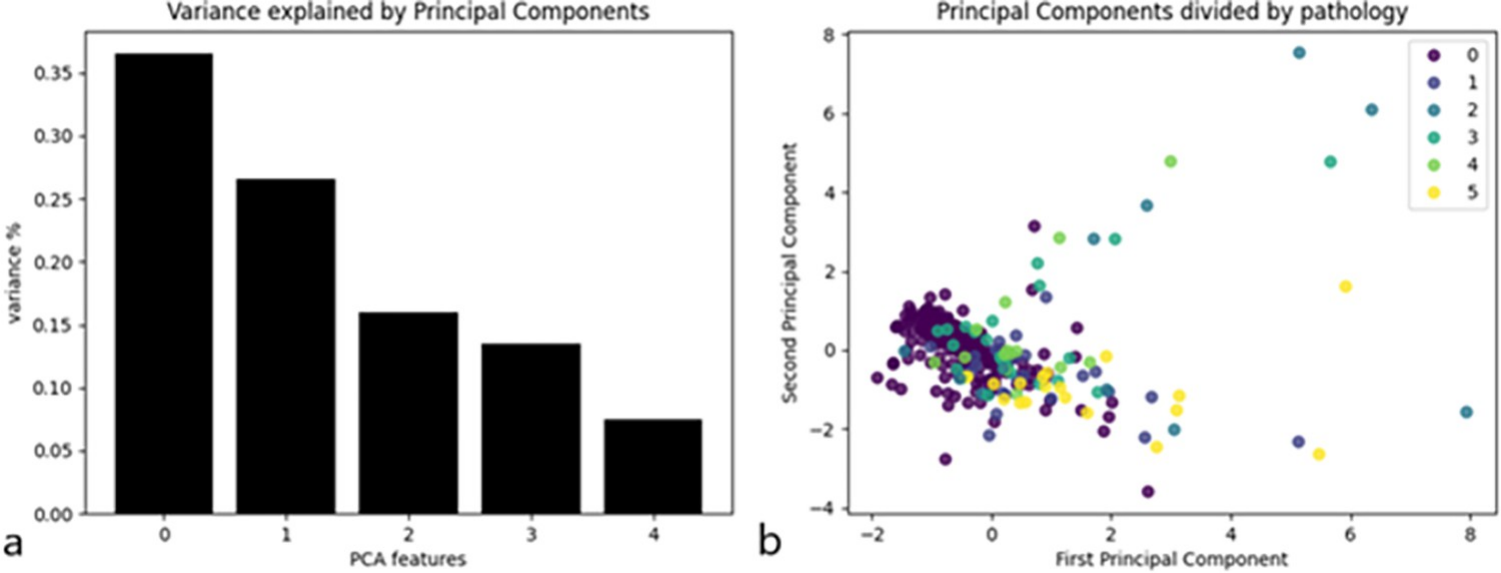
(a) Variance explained by principal components; (b) First and second principal component by pathology.

During the PCA, the variance of five principal components was analyzed, and the results are reported in Fig 11A. Since the first two principal components explain most of the variance, these were plotted in a scatterplot where the color legend represents the pathology (Fig 11B). This image shows that one cluster including most healthy subjects (label 0) can be found, while mostly pathological subjects are found outside the cluster.

## 4. Discussion

The aim of the present study was to propose and validate a new graphic test, SpAcCo, for assessing the dominant hand dexterity as a measure of fine motor disability. The SpAcCo execution requires little time (median time for healthy subjects per sheet = 59.4 s), it is feasible with inexpensive equipment (pen, paper sheet and digital timer), and does no need any prior training of the tested subjects. The SpAcCo test gives qualitative data since graphic track is visible and storable as well as quantitative data since execution time, number of touches or crossing and graphic trace length are calculated and recorded. The software analysis is based on traditional image analysis techniques, and the only required instrumentation to do it is a scanner and a computer with standard computational power.

We have validated the SpAcCo test in a healthy population of 200 subjects and we observed a performance decline with aging, as expected, showing that SpAcCo is sensitive enough to catch this aspect, already reported in the literature [19, 20]. Moreover, a positive learning effect was evident in the healthy cohort already at the second repetition of the graphic task although with a smaller width. These two features are specific for the healthy population but not for the pathological cohort of subjects. This is not surprising because subjects with a neurological disease have an increase “motor variability” due to an impairment of planning or execution skills [21]. Improving performance often means reducing action variability and it depends on calibration of motor system as well as central planning circuits [22]. These latter take advantage of reinforcement signals as well as experience-dependent plasticity.

SpAcCo rapresent a complex task requiring a degree of planning and sequencing movements, controlling force in grasping a pen and speed of drawing and therefore consistently integrating perceptual and motor information. Neurological diseases can affect any of this part or a combination and more patients need to be tested in order to establish if there is a specific disease pattern.

In fact, when compared, subjects with neurological disorders perform differently from healthy subjects in all total parameters (time, touches, crossings) but length. The comparison was performed by grouping all pathological subjects into one set, without distinction by pathology due to the limited numerousness of the subsets. In both cohorts exclusion criteria were low visual acuity not fully corrected by eyeglasses or contacts and cognitive impairment in order to minimize the effects on visual motor skills coordination [23].

The correlations between SpAcCo results and NHPT results in the case of pathological subjects show that not only the proposed graphic test provides comparable results with respect to the gold standard but is also able to discriminate between speed and precision. In fact, total time and number of errors are not correlated, which means that someone could be slow but precise, and someone else fast but imprecise. Given the correlation between time of execution and age, it is important to assess whether someone can complete the requested task, though slowly, or not. This feature cannot be understood by using only the NHPT, which only provides a time of execution of the task itself. Also, the NHPT test has a time limit so there is a known floor effect in case of patients that have extreme difficulties in completing the task, while the variety of information and the absence of time limit of the graphic test allow for a more thorough assessment of critical cases.

Another advantage of SpAcCo with respect to the NHPT is that the sheets and the digital copies remain in this case, so they can be consulted even after months or years, making it easier for the clinician to compare performances of the same patient, for instance before and after pharmacological treatment or physical therapy. Since the vast majority of patient try to compensate, at least in part, for the primary neurological deficit, by adopting new strategies, the SpAcCo test can be a valid outcome measure after rehabilitation since it provides more parameters that just the time execution.

In the present paper, there is no distinction between meander and spiral in the final computations, and this is because the number of patients is limited and with a wide variety of neurological diseases. However, this is a pilot study, and further data collection is required to specifically understand the characteristics of each group of patients. A kinematic analysis of the performance in the meander and in the spiral should be able to detect different types of difficulties. The meander requires rapid changes in direction, a task which is supposedly complex for ataxic patients [24] due to visual motor coordination impairment whereas the spiral requiring a continuing fluid movement is supposed to be more difficult for patients with extrapyramidal diseases and essential tremor [25]. These aspects need future studies with patient cohort more numerous and homogeneous. So far, it was possible to notice that, after the PCA, most healthy subjects belong to a cluster (Fig 11B), while mostly pathological subjects are found outside the cluster. This result suggests that a cluster analysis might provide an alert of the presence of a pathology. A complete cluster analysis should be carried out with bigger cohorts of pathological subjects to assess whether pathological clusters could also be identifiable.

A possibility to be explored in the future is to perform the test with an app instead of paper. This would allow to store automatically digitally all data, with no limit and to avoid intra- and inter-operator variability in taking the time. Also, all the standardization of different scanned files would no longer be a problem. In its actual form, first the tests must be performed, then the papers must be scanned, and the results reported manually in the case of times and analyzed with MATLAB® in the case of length, touches, and crossings. With an app, all parameters could be computed in real time and the results shown directly to the operator.

## Supporting information

S1 Data (XLSX)
